# Urinary Bladder Weight and Function in a Rat Model of Mild Hyperglycemia and Its Treatment With Dapagliflozin

**DOI:** 10.3389/fphar.2019.00911

**Published:** 2019-08-16

**Authors:** Zeynep Elif Yesilyurt, Betül Rabia Erdogan, Irem Karaomerlioglu, Ayhanim Elif Muderrisoglu, Martin Christian Michel, Ebru Arioglu-Inan

**Affiliations:** ^1^Department of Pharmacology, School of Pharmacy, Ankara University, Ankara, Turkey; ^2^Department of Pharmacology, Johannes Gutenberg University, Mainz, Germany

**Keywords:** diabetes, hyperglycemia, urinary bladder, hypertrophy, contraction, relaxation

## Abstract

Hypertrophy and dysfunction of the urinary bladder are consistently observed in animal models of type 1 and less consistently in those of type 2 diabetes. We have tested the effects of mild hyperglycemia (n = 10 per group) in a randomized, blinded study and, in a blinded pilot study, of type 2 diabetes (n = 6 per group) and its treatment with dapagliflozin (1 mg/kg per day) on weight, contraction, and relaxation of the rat bladder. Based on a combination of high-fat diet and a low dose of streptozotocin, animals in the main study reached a mean peak blood glucose level of about 300 mg/dl, which declined to 205 mg/dl at study end. This was associated with a small, if any, increase in bladder weight. In a pooled analysis of all animals of the main and the pilot study, we detected a correlation of moderate strength between blood glucose and bladder weight (*r* ^2^ = 0.2013; *P* = 0.0003 for Pearson correlation coefficient). Neither the main nor the pilot study found evidence for an altered contractility (responses to carbachol or KCl) or relaxation (responses to isoprenaline, fenoterol, CL 316,243, or forskolin). Treatment with dapagliflozin in the absence of hyperglycemia increased diuresis in the main study by 43% relative to control and increased bladder weight by 15% in the pooled groups of both studies (*post hoc* analysis). We conclude that mild hyperglycemia has no major effects on bladder hypertrophy or function.

## Introduction

More than 400 million people globally suffer from diabetes mellitus, and numbers are rising (World Health Organization, 2016). Most of them suffer from type 2 diabetes mellitus (T2DM). Lower urinary tract dysfunction, in general, and bladder dysfunction in particular are estimated to occur in 80% and 50% of all diabetic patients, respectively (Daneshgari and Moore, 2006). For instance, we have previously shown that men with concomitant diabetes exhibit lower urinary tract dysfunction equivalent to that in men without diabetes but 12 years older (Michel et al., 2000). The mechanisms leading to bladder dysfunction in diabetic patients are only incompletely understood.

Injection of streptozotocin (STZ) is the most frequently used animal model of type 1 diabetes mellitus (T1DM), and STZ-injected rats consistently exhibit bladder hypertrophy, by average, a doubling of bladder weight (Arioglu Inan et al., 2018). Despite the much greater incidence of T2DM as compared with T1DM, only a few studies have assessed bladder weight in models of T2DM. Moreover, these models have yielded heterogeneous results with some models, such as Zucker diabetic fatty rats, exhibiting as much hypertrophy as STZ-injected rats, but others, such as db/db mice, exhibiting little bladder enlargement (Ellenbroek et al., 2018).

Against this background, our study was designed to test the hypothesis that urinary bladder hypertrophy occurs in a frequently used model of T2DM, a combination of high-fat diet (HFD) with low-dose STZ injection (Daneshgari et al., 2009). As a secondary and explorative aim, we have assessed bladder function by measuring contraction and relaxation of isolated bladder strips in response to agonists at muscarinic receptors and β-adrenoceptors, respectively, and receptor-independent stimuli.

One hypothesis explaining diabetes-associated bladder hypertrophy assumes that the hyperglycemia leads to polyuria, which then leads to hypertrophy (Turner and Brading, 1999). However, a weak correlation between mean blood glucose concentrations and extent of bladder hypertrophy in STZ-injected rats does not support the polyuria hypothesis as a major cause of hypertrophy (Arioglu Inan et al., 2018). Insulin treatment can reverse bladder hypertrophy in the rat STZ model (Longhurst et al., 1991; Fukumoto et al., 1994; Xiao et al., 2015), but it is unclear whether treatments typically used in T2DM also can do so. An emerging drug class for the treatment of T2DM is sodium glucose cotransporter 2 (SGLT2) inhibitors, such as dapagliflozin and empagliflozin (Vallon, 2015). SGLT2 inhibitors promote glucose excretion and, thereby, increase diuresis (Michel et al., 2015). Accordingly, an SGLT2 inhibitor may on the one hand cause bladder enlargement by enhancing diuresis; on the other hand, it may ameliorate diabetes-associated enlargement by improving glucose control. Therefore, it has been another pre-specified, hypothesis-testing aim of our study to determine whether the SGLT2 inhibitor dapagliflozin reverses bladder hypertrophy.

## Methods

### Animals and Treatments

We have used animals that had been generated as part of a study to explore effects of the treatments on the heart (to be reported elsewhere). This underlying study was performed in two batches of 6 and 10 animals per group, respectively. For the purpose of the functional experiments of the bladder project, we considered the first batch as a pilot study and the second batch as the main study. The study protocol had been approved by the animal welfare committee of Ankara University (permit 2015-4-82, 2017-9-84, 2017-23-188) and was in line with NIH Guidelines for Care and Use of Laboratory Animals.

Male Sprague Dawley rats (5 weeks old) were obtained from Bilkent University Genetics and Biotechnology Research Center (Ankara, Turkey) and housed under 12:12 h lighting conditions with free access to chow and tap water. After 1 week of quarantine, rats received either standard chow [Purina Rat Chow (5% fat); Optima AS, Bolu, Turkey] or HFD [OpenSource diet, D12492 (35% fat); Arden Research & Experiment, Ankara, Turkey] for the rest of the study. After another 4–5 weeks, animals of the HFD group received an intraperitoneal injection of STZ (25 mg/kg dissolved in citrate buffer at pH 4.5) or vehicle. Animals with blood glucose levels <200 and <140 mg/dl in batches 1 and 2, respectively, received a second or, if necessary, third STZ injection. After diabetes had been established, i.e., 18 to 21 weeks of age (a minimum of 6 weeks after STZ injection), half of control and HFD + STZ rats started receiving daily oral treatment with dapagliflozin (1 mg/kg per day, Online **Supplemental Figure 1**) or vehicle. Dapagliflozin suspension was made in distilled water after powdering of Forziga^®^ tablets (10 mg as 12.3 mg dapagliflozin propanediol monohydrate). The tablets included microcrystalline cellulose, anhydrous lactose, crospovidone, silicon dioxide, and magnesium stearate as pharmaceutically inactive ingredients. Their film coating contains polyvinyl alcohol, titanium dioxide, polyethylene glycol, talc, and yellow iron oxide. These daily treatments were continued until study end (for technical reasons 13–15 and 12–13 weeks after the start of treatment with dapagliflozin in batches 1 and 2, respectively). Thus, our study had four arms (based on randomization for the second batch): group I was healthy controls, group II received HFD and low-dose STZ (HFD/STZ), group III received dapagliflozin (dapa), and group IV HFD plus low-dose STZ plus dapagliflozin (HFD/STZ/dapa). Randomization was applied at the time point when treatment with dapagliflozin or vehicle started for entry into the *in vitro* bladder experiments, i.e., between animals without (control vs. HFD/STZ) and with treatment (dapa vs. HFD/STZ/dapa). For unforeseen reasons, more rats reached the planned time point for the *in vitro* experiments than what could technically be handled in batch 2; when this occurred, we first studied the control and HFD/STZ rats and then the dapa and HFD/STZ/dapa rats leading to a longer period from randomization to euthanasia for the latter two groups.

**Figure 1:**
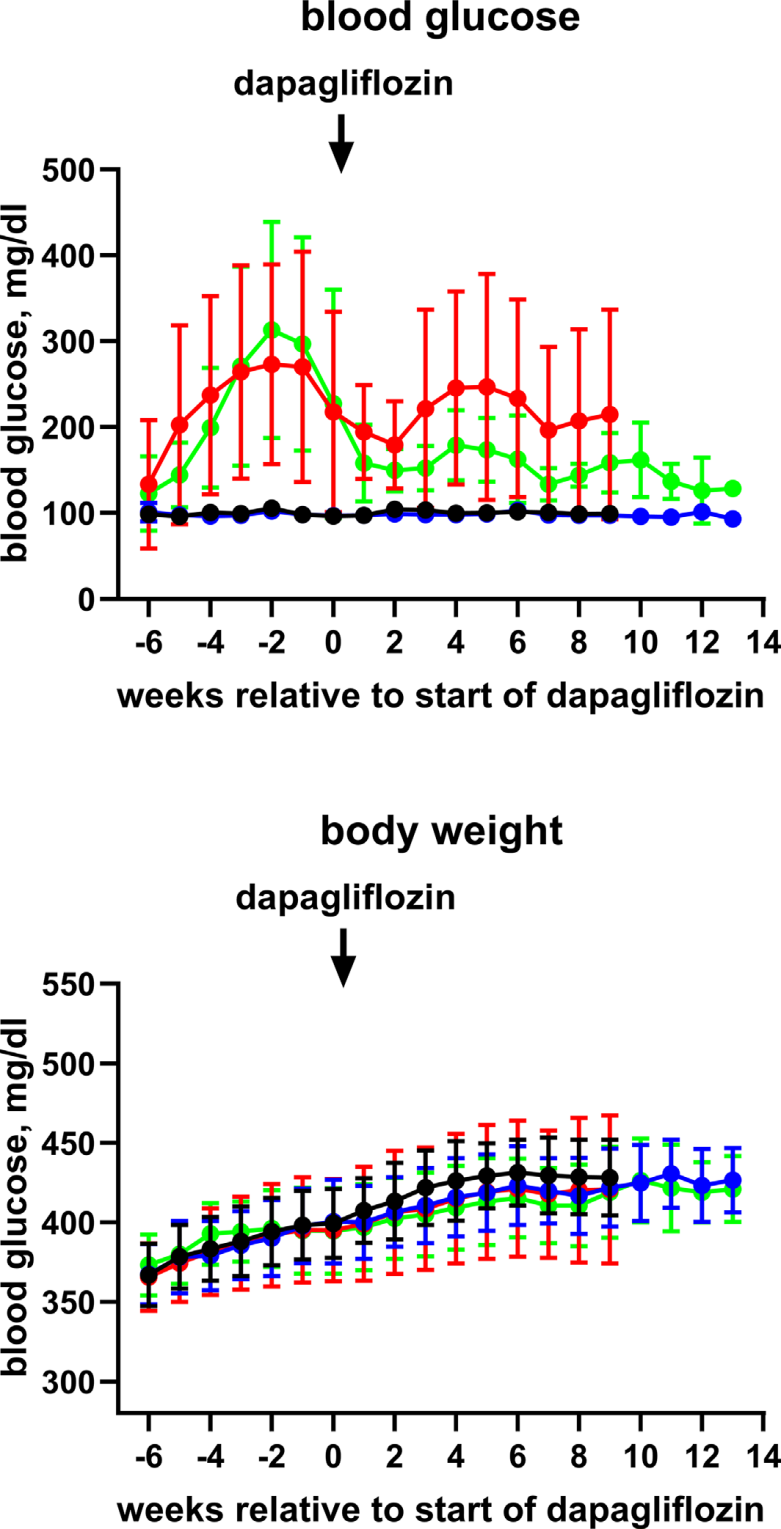
Time course of blood glucose and body weight of control rats (black symbol), control rats treated with dapagliflozin (blue symbols), HFD + low-dose STZ-treated rats (red symbols) and HFD + low-dose STZ + dapagliflozin-treated rats (green symbol). Each data point represents mean ± SD of 10 rats (9 for HFD + low-dose STZ group). As start of treatment with dapagliflozin differed slightly between animals, data are shown relative to the individual start of treatment (weeks 20–21).

Blood glucose (contour plus glucose test strips) and body weight were monitored weekly (**Figure 1** and **Supplementary Figure 1**; due to minor difference in absolute age between animals, data in these figures are shown relative to start of treatment with dapagliflozin). End-of-study measurements of blood glucose and body weight were made during the last week before the *in vitro* experiments. During that week, we also placed rats from batch 2 individually in metabolic cages for 24 h to determine the food and water intake and feces and urine output. Animals were killed by exsanguination under anesthesia with inhalation of 2% isoflurane (batch 1) or ether (batch 2).

### Organ Bath Experiments

The urinary bladder was excised, freed from adjacent adipose and soft connective tissue, and weighed. After removal of the upper most dome and the lower trigone area, the remaining body of the bladder was cut into four longitudinal strips of approximately 1 to 2 mm width, 16.8 ± 4.2 mm length and weighing 19.0 ± 6.8 mg. Based on previous validation experiments (Schneider et al., 2011), strips were stored in ice-cold Krebs-Henseleit buffer for up to 2 h prior to use in the organ bath.

Organ bath experiments were performed as described previously (Michel, 2014) with minor modifications. Briefly, strips were mounted in a 10-ml organ bath in Krebs-Henseleit buffer (118.5 mM NaCl, 4.7 mM KCl, 1.2 mM MgSO_4_, 2.5 mM CaCl_2_, 1.2 mM KH_2_PO_4_, 25 mM NaHCO_3_, and 5.6 mM glucose continually gassed with 95% O_2_/5% CO_2_ to maintain a pH of 7.4 at 37°C) under a resting tension of 10 mN. They were allowed 75 min of equilibration, including washes with fresh buffer every 15 min and re-adjustment of resting tension after each wash. Each strip was challenged twice with 50-mM KCl (maintaining iso-osmolarity by reducing NaCl concentration from 116.8 to 68.5 mM) with 60-min rest between challenges; the peak response to the second KCl addition was used to describe receptor-independent contraction. After washing and an additional 45-min equilibration, a carbachol concentration-response curve was generated by adding cumulative concentrations of the muscarinic agonist carbachol (10 nM to 300 μM) in half-logarithmical steps, and peak tension was measured for each concentration. After the highest carbachol concentration, strips were washed and allowed another 45 min of equilibration. Thereafter, 1-µM carbachol was added. When steady-state tension was reached, a cumulative concentration-response curve for a β-adrenoceptor agonist was generated in half-logarithmical steps every 4–5 min (isoprenaline, 0.3 nM to 30 μM; fenoterol, 0.03 nM to 30 μM; CL 316,243, 0.1 nM to 3 μM). After the peak response to the highest concentration of receptor agonist had been observed, 10-µM forskolin was added to assess receptor-independent relaxation. At the end of the experiment, strips were removed from the organ bath and strip length, and weight was measured.

### Chemicals

Dapagliflozin tablets (Forziga^®^) were obtained from Astra Zeneca (Ankara, Turkey), crushed in a mortar and suspended in distilled water. The materials for Krebs solution, STZ, carbachol, CL 316,243 (disodium(R,R)-5-[2-[2-(3-chlorophenyl)-2-hydroxyethyl]-amino]propyl]-1,3-benzodioxole2,2-dicarboxylate), fenoterol, and isoprenaline were obtained from Sigma Aldrich (Darmstadt, Germany). Isoflurane and diethyl ether were from Adeka (Samsun, Turkey) and from Merck (Billerica, MA, USA), respectively. Glucose test strips (Contour Plus) were from Bayer (Ankara, Turkey).

### Data Analysis and Quality Measures

The following sample size considerations were pre-specified: all available animals from the underlying heart study were used for blood glucose, bladder weight, and body weight (pre-specified sample size n = 16). A sample size of n ≥ 8 was specified for the exploratory organ bath experiments of the main study (batch 2); this sample size was chosen based on the observed variability in our previous studies on rat bladder relaxation by β-adrenoceptor agonists (Schneider et al., 2004; Frazier et al., 2005; Schneider et al., 2005a; Michel, 2014). One of the animals in the diabetic group of the second batch died; it was not replaced. Therefore, this group comprises only nine animals. Group allocation in the second batch (main study) was randomized. All experiments and analyses of both batches related to bladder weight and function were performed with blinding to group allocation; blinding included not only concealment of group allocation but also of blood glucose and body weight because the latter may have indirectly unblinded the assessment. Unblinding was performed after all experimental data of a given batch have been analyzed.

Concentration-response curves for carbachol and the β-adrenoceptor agonists were analyzed by fitting sigmoidal curves to the experimental data; these fitted top, bottom, and pEC_50_ of the curve under the assumption of a Hill slope of 1. Based on previous studies (Schneider et al., 2004; Schneider et al., 2005b), we were aware that contractile responses to carbachol typically reach a maximum at 30 µM of the agonist with smaller contractions at higher concentrations, i.e., exhibit a bell-shaped curve. Because the nature of the declining part of the concentration-response curve is unclear, we pre-specified to analyze only responses for 10 nM to 30 µM in the control group. However, we also tested 100 and 300 µM carbachol in all groups to allow detection of possible right shifts of the curve upon treatment. As we observed a decline of contractions at 100 and 300 µM carbachol in every single rat of all treatment groups, we also restricted curve fitting in the treated groups to the 10-nM to 30-µM range, which is in line with our previous studies (Frazier et al., 2007; Michel, 2014). We have previously reported that normalization of contraction data to strip length reduces the coefficient of variation (Schneider et al., 2005a). Therefore, this normalization was pre-specified for the present study. However, we used all strips tested in the present study to validate this normalization in an independent data set (Erdogan et al., 2019). As these data show that normalization is more effective when strip weight is used as the denominator, we also performed an analysis after normalization of contraction data for strip weight (shown in **Online Supplement**). Relaxation data were normalized with the plateau response to 1-μM carbachol as measured immediately prior to the addition of relaxing agonists set as 100% and basal force as measured prior to addition of carbachol set as 0%.

Some interventions were typically assessed in more than one strip from a given rat (KCl, carbachol and forskolin mostly in four strips, isoprenaline mostly in two strips). In these cases, the average of all technical replicates from one rat was considered as n = 1.

The primary aim of the study was to test whether bladder hypertrophy assessed as bladder weight exists in HFD/STZ rats and whether treatment with dapagliflozin ameliorates this (null hypothesis: no change). To preserve statistical α, this was assessed by hierarchical testing. In the first step, we determined whether bladder weight was greater in the HFD/STZ animals than in the control group. In the second step, it was tested whether it was smaller in the HFD/STZ/dapa than in the HFD/STZ group; this second step was only performed if the first step indicated a statistically significant difference. These null hypotheses were tested by unpaired, two-sided *t*-tests with statistical α set at 0.05. The last measured glucose and body weight value was used for the exploration of a relationship between extent of glucose elevation and bladder weight.

After completion and unblinding of the study, we realized that the HFD/STZ group of batch 1 exhibited major increases in end-of-study blood glucose levels (**Supplemental Table 1**), whereas batch 2 had only a minor increase (**Table 1**). This led to the *post hoc* decision not to pool results from both batches but rather to analyze them separately (except for correlation analysis between blood glucose and bladder weight). Therefore, we primarily report here the findings from the main study (batch 2) as a condition of mild hyperglycemia and show those of batch 1 exhibiting diabetes in the **Online Supplement**.

**Table 1:** Physiological parameters prior to sacrifice.

	Control	Dapagliflozin	HFD/STZ	HFD/STZ/dapa
Blood glucose, mg/dl	100 ± 7	99 ± 7	205 ± 118	130 ± 31
Body weight, g	430 ± 23	430 ± 22	424 ± 44	421 ± 20
Bladder weight, mg	161 ± 33	183 ± 18	175 ± 46	192 ± 11
Bladder/body weight, g/100 g	0.30 ± 0.16	0.43 ± 0.04	0.43 ± 0.15	0.46 ± 0.04

Data are means ± SD of 10 rats, except for HFD/STZ group (n = 9) as based on the last measurement before study end. Bladder weight did not differ significantly between HFD/STZ and control animals (null hypothesis rejected). Note that some values differ from the last data point in **Figure 1** because end-of-study measurement was not in the same week for all rats.

All parameters other than bladder weight and all group comparisons other than healthy versus HFD/STZ and HFD/STZ vs. HFD/STZ/dapa were considered as exploratory endpoints. Therefore, no hypothesis-testing statistical analysis was applied to those parameters and groups; rather, means ± SD are reported. These included blood glucose and body weight in all groups and bladder/body weight ratio and contractile and relaxation responses in the organ bath experiments. All curve fitting and statistical analyses were performed with Prism (version 7.1; Graphpad, La Jolla, CA, USA).

## Results

### Physiological Parameters

Blood glucose levels reached values >400 mg/dl in the HFD/STZ-treated groups prior to the start of treatment with dapagliflozin in batch 1 and remained at that level until end of treatment (Online **Supplemental Figure 2**). In batch 2, however, peak mean glucose concentrations in the HFD/STZ-treated groups were only 270 to 310 mg/dl and spontaneously declined to about 200 mg/dl before the start of treatment with dapagliflozin (**Figure 1**). They remained at that level until end of treatment (**Table 1**). Although treatment with dapagliflozin had no major effect on glucose levels in control animals, it further reduced them to about 130 mg/dl in HFD/STZ/dapa rats (**Figure 1**; **Table 1**). Because end-of-treatment glucose levels differed considerably between batches, it was decided *post hoc* that pooling of both batches was inappropriate. Accordingly, we here report only data from batch 2 and disclose those of batch 1 in the **Online Supplement**.

**Figure 2:**
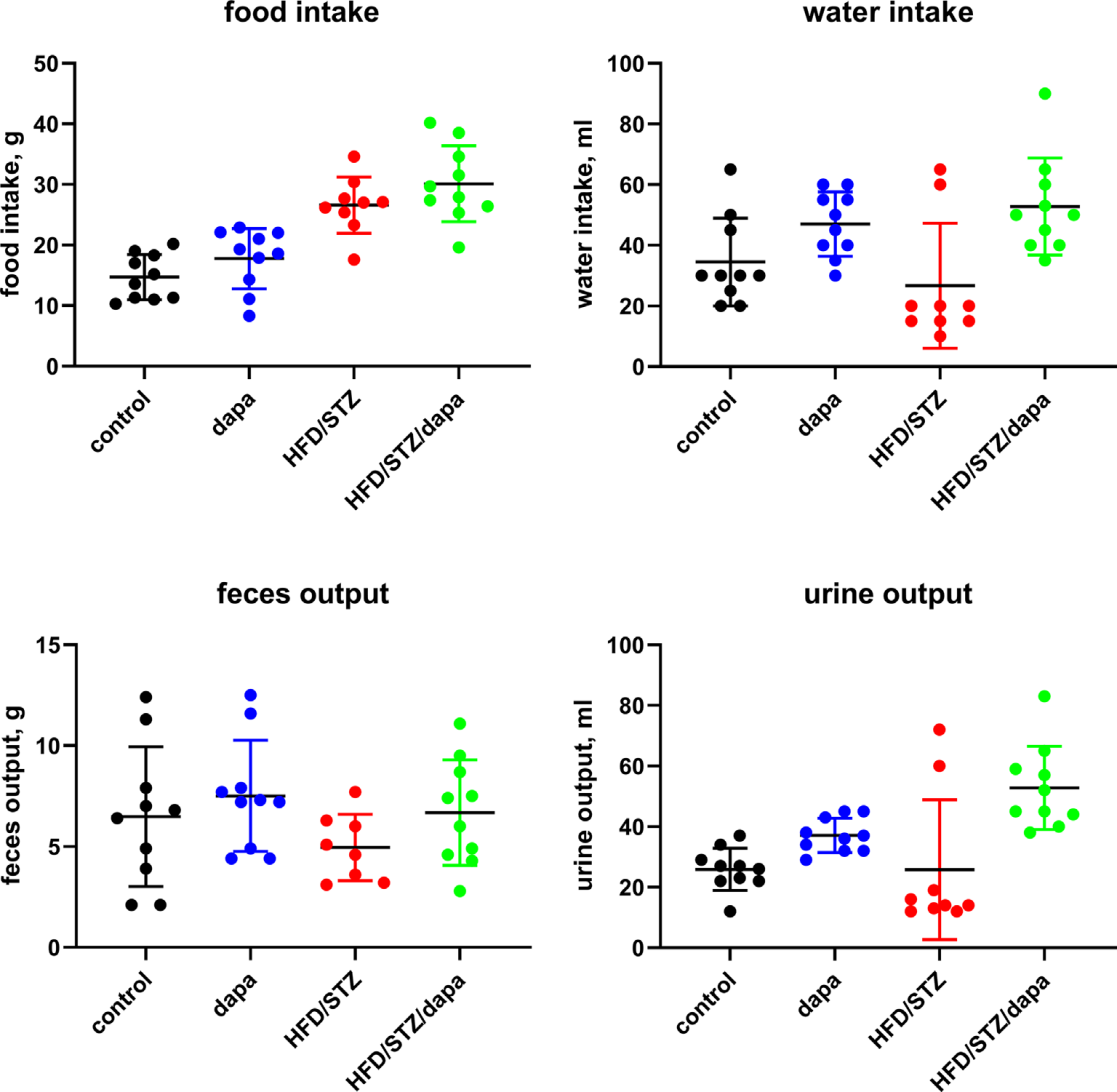
Food and water intake and feces and urine output per 24 h as measured in metabolic cage experiments during the last week prior to sacrificing. Each data point represents data from one animal and the horizontal lines group means ± SD.

Body weight developed similarly over time in all groups (**Figure 1**) and was comparable at the end of treatment (**Table 1**). In metabolic cage experiments during the last week prior to sacrifice, food intake was greater in HFD/STZ than that in the control rats with little effect of concomitant treatment with dapagliflozin (**Figure 2**). Feces output was comparable across all four groups. In contrast, water intake and urine output were similar in control and HFD/STZ rats but increased in those additionally receiving treatment with dapagliflozin (37 ml vs. 26 ml in dapa vs. control, mean difference 11 [95% confidence limit 5; 17] ml; **Figure 2**).

Bladder weight, the primary endpoint of the study, also was comparable between all four groups (**Table 1**). In a direct comparison between the HFD/STZ and the control group, no statistically significant difference was observed (*P* = 0.5053 in two-tailed, unpaired *t*-test, mean difference with 95% confidence interval, 13.4 [-27.4; 55.2] mg, null hypothesis not rejected). Therefore, based on our chosen strategy of hierarchical testing, no additional statistical testing was performed. The bladder/body weight ratio was also comparable between groups (**Table 1**). In a *post hoc* comparison of the pooled data from both batches suggested by one of the reviewers, bladder weight was slightly greater in the dapa as compared with the control group [182 vs. 157 mg, descriptive *P* = 0.0083 in a two-tailed, unpaired t-test; mean difference, 24 (7; 42) mg].

**Figure 3:**
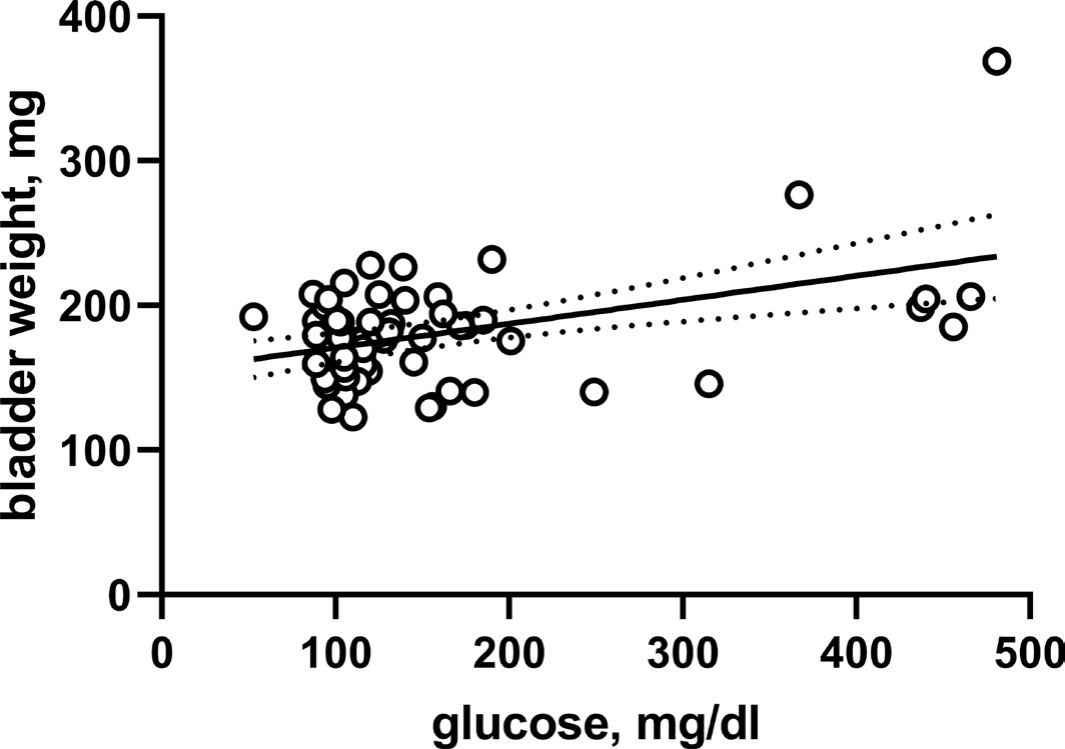
Correlation between bladder weight and glucose across both batches and all experimental groups. Each data point represents a single animal, the solid line the regression line and the dotted lines the 95% confidence intervals thereof.

The HFD/STZ rats of batch 1 also did not exhibit major bladder hypertrophy (**Supplemental Table 1**). However, comparison of intra-individual glucose and bladder weight data from the pooled animals of both batches yielded a weak (r^2^ = 0.2013) but statistically significant correlation (*P* = 0.0003 for deviation of Pearson correlation coefficient from 0; **Figure 3**). Of note, this correlation was driven by seven animals with blood glucose levels >300 mg/dl at study end including six rats from batch 1.

### Contraction Experiments

**Figure 4:**
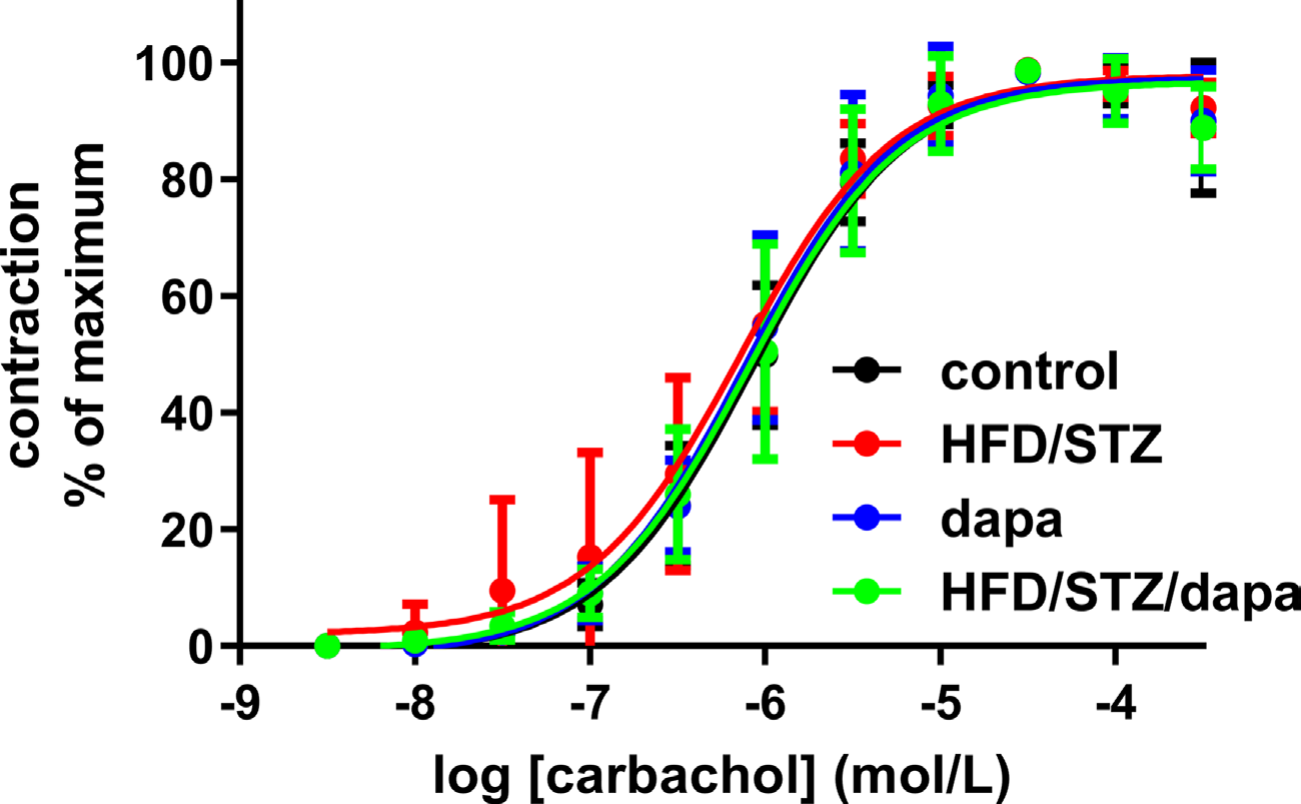
Contractile responses to concentration-response curve of carbachol (peak responses). Data are shown as mean ± SD of 10 animals, except for hyperglycemic group (n = 9) and have been expressed as % maximum response within an experiment. Quantitative data are shown in **Table 2.**

Contractile responses were assessed in an explorative manner in three ways, as peak response to 50 mM KCl, as E_max_ and pEC_50_ for carbachol derived from the concentration-response curves for peak responses, and as plateau response to the carbachol concentration given immediately prior to start of the concentration-response curves for the β-adrenoceptor agonists. Based on the pre-specified protocol, contraction was analyzed upon normalization for strip length. All four indices of bladder contractility were comparable across groups (**Table 2**, and **Figure 4**). This was also true when contraction data were normalized for strip weight in a *post hoc* analysis (**Supplemental Table 2**). Contractile responses were also comparable across groups in the animals of batch 1, despite exhibiting a more profound increase in blood glucose (**Supplemental Table 3**).

**Table 2:** Contractile responses of bladder strips.

	Control	Dapagliflozin	HFD/STZ	HFD/STZ/dapa
KCl peak, mN/mm	294 ± 98	337 ± 116	316 ± 79	365 ± 105
Carbachol peak pEC_50_	6.00 ± 0.20	6.04 ± 0.24	6.20 ± 0.47	5.99 ± 0.32
Carbachol peak E_max_, mN/mm	548 ± 198	693 ± 258	588 ± 166	763 ± 218
Carbachol plateau, mN/mm	159 ± 47	188 ± 53	170 ± 38	189 ± 55

Data are means ± SD of 10 rats, except for HFD/STZ group (n = 9) with 2-4 strips being measured per rat. A graphical depiction of the data is shown in **Figure 4**.

### Relaxation Experiments

**Figure 5:**
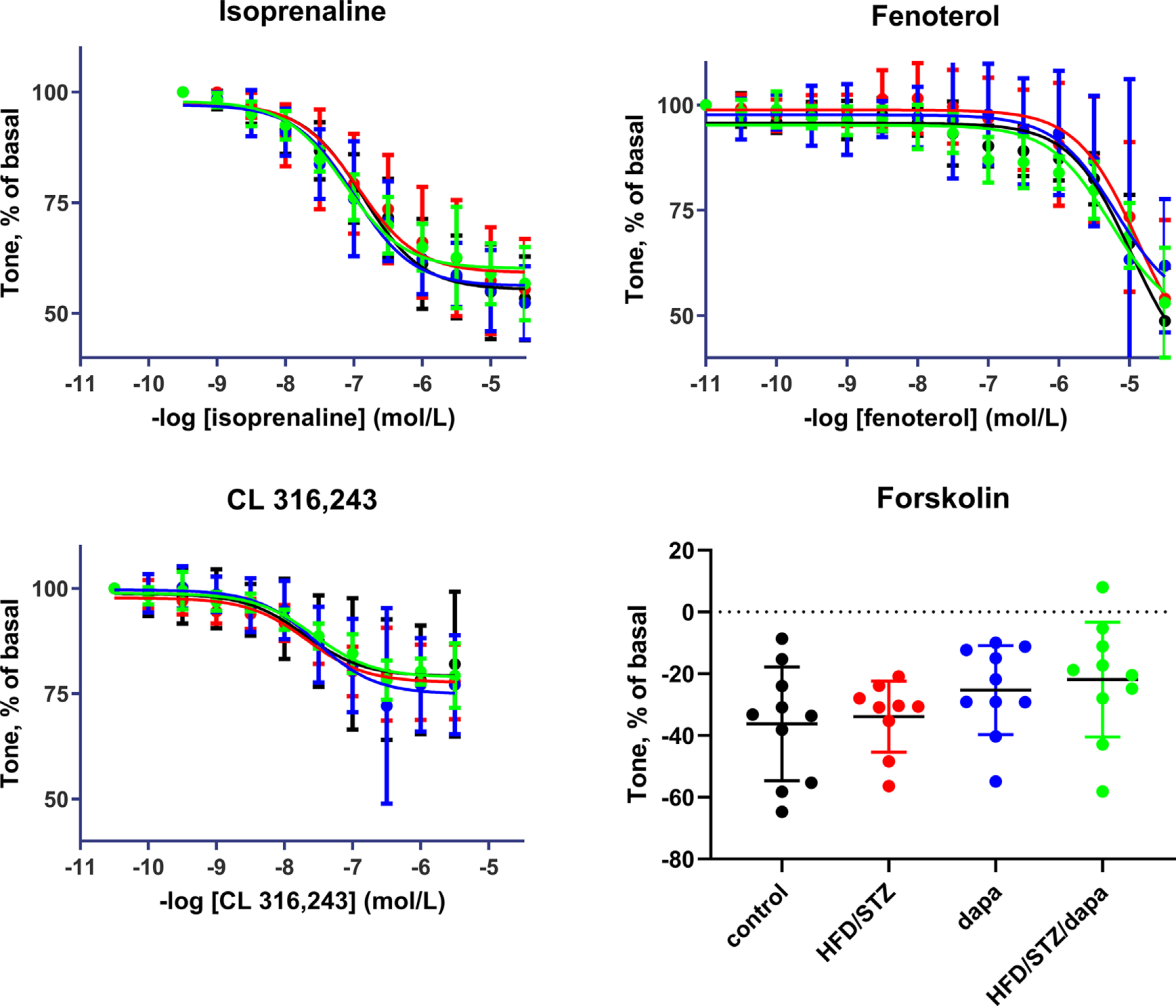
Relaxation responses to the general β-adrenoceptor agonist isoprenaline, the β_2_-adrenoceptor-selective agonist fenoterol, the β_3_-adrenoceptor-selective CL 316,243 in control (black), hyperglycemic (red), dapagliflozin-treated controls (blue) and dapagliflozin-treated hyperglycemic animals (green), and, as receptor-independent relaxant stimulus for forskolin (10 µM). Data are means ± SD of 10 experiments (9 only for hyperglycemic group) for the agonists; for forskolin, each data point is the mean of 1 to 3 replicates in a given rat. Quantitative data are shown in **Table 3**.

Relaxation responses were assessed in an explorative manner for the general β-adrenoceptor agonist isoprenaline, the β_2_-adrenoceptor-selective agonist fenoterol, the β_3_-adrenoceptor-selective agonist CL 316,243, and, as receptor-independent relaxant stimulus, for forskolin. While a full concentration-response curve was generated for each of the β-adrenoceptor agonists, forskolin was tested in a single concentration of 10 μM. Potency and efficacy were comparable across the four treatment groups for all three agonists of batch 2, as was the effect of the single forskolin concentration (**Table 3**, and **Figure 5**). Similarly, they were comparable across groups in batch 1 (**Supplemental Table 4**). Although inspection of the fenoterol data clearly showed that responses were similar across groups in both batches of animals, the individual curves did not reach a clear maximum effect within the tested concentration range in several cases. This resulted in fitted fenoterol curves yielding estimates of pEC_50_ and E_max_ extending beyond the measured values in some cases, raising questions on the validity of these estimates. Concomitantly, there was considerably greater variability of estimates of pEC_50_ and E_max_ of fenoterol between experiments than for the other agonists. Therefore, we additionally quantified the relaxation response to the highest fenoterol concentration (30 μM) in a *post hoc* analysis; this response was also comparable across groups (**Table 3**, **Supplemental Table 4**).

**Table 3:** Relaxant responses of bladder strips.

	Control	Dapagliflozin	HFD/STZ	HFD/STZ/dapa
Isoprenaline				
pEC_50_	6.9 ± 0.3	7.1 ± 0.4	6.9 ± 0.4	7.2 ± 0.2
E_max_	56 ± 9	56 ± 6	59 ± 11	60 ± 8
Fenoterol				
pEC_50_	5.2 ± 0.3	6.2 ± 1.6	5.9 ± 1.6	5.9 ± 0.5
E_max_	41 ± 15	65 ± 26	54 ± 28	58 ± 14
30 µM	49 ± 13	62 ± 16	54 ± 19	53 ± 13
CL 316,243				
pEC_50_	7.8 ± 0.2	7.6 ± 0.1	7.9 ± 0.3	7.6 ± 0.2
E_max_	80 ± 15	74 ± 8	78 ± 9	79 ± 5
Forskolin				
10 µM	-36 ± 18	-25 ± 14	-34 ± 11	-22 ± 19

Data are means ± SD of 10 rats, except for HFD/STZ group (n = 9) with 1 strip being measured per rat for the *β*-adrenoceptor agonists and the mean of 1-3 strips for forskolin. Maximum relaxation responses (E_max_) and those to 10 µM forskolin are expressed as % of tone, i.e. force prior to addition of first agonist concentration (“carbachol plateau”, see **Table 2**). A graphical depiction of the data is shown in **Figure 5**.

## Discussion

### Critique of Methods

Our study was designed to investigate bladder hypertrophy in a rat model based on a combination of HFD and low-dose STZ injection. This combination is a routinely applied T2DM model (Zhang et al., 2012; Lee et al., 2018; Li et al., 2018; Liu et al., 2018). Although increased body weight is a typical feature of human T2DM, this is not mimicked by the HFD/STZ model (Zhang et al., 2012; Lee et al., 2018; Li et al., 2018; Liu et al., 2018) as confirmed in the present study. Therefore, our model does not recapitulate the obesity phenotype typically seen in T2DM and may include elements of a T1DM phenotype. Studies in STZ-injected rats have demonstrated that the development of bladder hypertrophy is largely complete after 1 week of induction (Arioglu Inan et al., 2018).

For reasons of efficient and ethical use of experimental animals, our experiments have used surplus tissue from an ongoing study on cardiac effects of T2DM. This study was performed in two batches based on the needs of the cardiac investigations. Our original study protocol specified to pool data from both batches for analysis of bladder enlargement but to use the first batch as a pilot and the second larger batch as the main study for the organ bath experiments. Both batches had a largely identical design. Accordingly, the HFD/STZ group of both batches exhibited similar peak increases in blood glucose to about 400 and 300 mg/dl in batches 1 and 2, respectively. Although glucose level remained stable at that level in batch 1, they declined over time in batch 2, reaching pre-dapagliflozin and end-of-study levels of about 200 mg/dl. Although the end-of-study glucose level in batch 1 was in line with reports of others in this model (Zhang et al., 2012; Lee et al., 2018; Li et al., 2018; Liu et al., 2018), those of batch 2 were not and probably cannot be considered diabetic but rather as mildly hyperglycemic. We consider a partial recovery of pancreatic beta cells after a less effective destruction by STZ (Desgraz et al., 2011; Cheng et al., 2016) as the most likely explanation for the decline of blood glucose in batch 2. Studies in the rat STZ model of T1DM have demonstrated that treatment with insulin can largely reverse diabetes-associated bladder hypertrophy within a few weeks (Longhurst et al., 1991; Fukumoto et al., 1994; Xiao et al., 2015). As glucose levels were considerably <300 mg/dl for at least 10 weeks prior to euthanasia in the HFD/STZ group of batch 2, bladder weight most likely reflects these mildly hyperglycemic and not the early diabetic levels observed over the time course of our study. Therefore, a *post hoc* decision was made not to pool data from batches 1 and 2 to test the effects of diabetes and treatment with dapagliflozin on bladder weight and function. Of note, our treatment with dapagliflozin started at least 6 weeks after the first STZ injection, i.e., when bladder enlargement is expected to have fully developed (Arioglu Inan et al., 2018), thereby giving our study a treatment and not a prevention design.

It was a co-primary aim of our study to test the effect of dapagliflozin on bladder weight in HFD/STZ rats. Our treatment with dapagliflozin was not based on pure chemical but on crushed tablets of the clinically used formulation. Although this may be weaker evidence specific for the dapagliflozin molecule, it is closer to the clinical situation, thereby enhancing translational value. Our metabolic cage experiments validate this approach and the efficacy of oral dosing by demonstrating that the dapagliflozin-treated groups had a greater urine output (and correspondingly greater fluid intake) than the control and HFD/STZ groups, as typical for treatment with an SGLT2 inhibitor (Michel et al., 2015).

Hypertrophy studies in other tissues often normalize tissue for body weight. As discussed in detail elsewhere (Arioglu Inan et al., 2018; Ellenbroek et al., 2018), both T1DM and T2DM often are associated with changes in body weight. If bladder hypertrophy is assessed as bladder/body weight ratio, changes may occur that are at least partly driven by those of the denominator body weight. Nonetheless, it remains unclear whether bladder weight or bladder/body weight is the more informative indicator of bladder hypertrophy in diabetes models. When designing our study, we had decided to make bladder weight the primary outcome parameter, but to report bladder/body weight concomitantly for full transparency. In line with our primary interest in the effects of dapagliflozin on bladder hypertrophy, we had pre-specified a strategy of hierarchical statistical testing. As bladder weight did not differ markedly between control and the HFD/STZ group, no statistical comparison between the latter and the dapagliflozin-treated group was made.

Several measures were implemented to assure data quality: First, treatment allocation had been randomized for batch 2 (comparison of control vs. HFD/STZ and dapa vs. HFD/STZ/dapa), and all experiments and analyses for both batches were performed under blinded conditions for treatment allocation. Unblinding was performed after completion of all analyses for a batch and data base lock. Second, although sample size was driven by the choices made by the cardiac investigations, we had pre-specified for our analysis to include all bladders from both batches for the primary outcome parameter, bladder weight. However, because the second batch did not develop diabetes, we have made a *post hoc* change in our analysis strategy to analyze batch 1 and batch 2 separately at the level of group means, but to pool data from both batches for the correlation analysis with blood glucose. Moreover, sample size in batch 1 is in the range of what other studies in the bladder hypertrophy field have reported, and that in batch 2 markedly exceeds sample size in most other studies (Arioglu Inan et al., 2018; Ellenbroek et al., 2018). Third, our study followed a pre-specified statistical analysis plan. This has applied hypothesis-testing statistics only for the pre-specified primary outcome variable (bladder weight). To maintain statistical α, hierarchical testing was applied. All other experimental outcomes were considered exploratory. In line with recent recommendations from more than 800 experts in the field of statistical data analysis (Amrhein et al., 2019), we only applied descriptive statistics for those parameters and focused on reporting of effect sizes with 95% confidence intervals.

To address our exploratory secondary aim of assessing bladder contractility, we have chosen a three-pronged approach by studying full concentration-response curves for carbachol based on peak responses to each agonist concentration, the plateau contraction response to a single concentration of carbachol, and the peak contraction response to KCl as receptor-independent stimulus. All three approaches yielded comparable results for effects of HFD/STZ versus control and lack of effect of additional treatment with dapagliflozin. Based on previous experience (Schneider et al., 2005a; Schneider et al., 2011), we had pre-specified to analyze contraction data after normalization for strip length. However, a secondary analysis of the present data set suggested that normalization for strip weight may reduce variability more than normalizing for length (Erdogan et al., 2019), indicating that strip weight is a more informative denominator for contraction data. Therefore, we not only report data primarily with normalization for strip length as pre-specified in our study design but also report them normalized for strip weight in the **Online Supplement**.

To explore relaxation responses, we had to consider that relaxation of rat bladder is known to involve a mix of β_2_-and β_3_-adrenoceptors, whereas it occurs predominantly by β_3_ adrenoceptors in the human bladder (Michel and Vrydag, 2006). Therefore, we have used isoprenaline as a general β-adrenoceptor agonist, fenoterol is one of the most selective β_2_-adrenoceptor agonists (Hoffmann et al., 2004; Baker, 2010), and CL 316,243 as one of the most selective agonists for the rodent β_3_ adrenoceptor (Cernecka et al., 2014). After completion of the experiments, we were surprised by the low potency of fenoterol in both batches and became aware that one group had proposed that fenoterol acts *via* β_3_-adrenoceptors in rat bladder (Palea et al., 2012). However, the low potency of fenoterol could also be explained by the general observation that β-adrenoceptor agonists become less potent and less efficacious when relaxation is tested against contraction induced by a muscarinic receptor agonist (Cernecka et al., 2014; Dale et al., 2014). Although we cannot exclude based on the present data that fenoterol acted at least partly *via* β_3_-adrenoceptors, it remains as one of the most selective β_2_-adrenoceptor agonists (Hoffmann et al., 2004; Baker, 2010). Therefore, we consider it likely that the observed fenoterol responses reflect those of β_2_-adrenoceptor stimulation but cannot exclude that they at least partly also reflect β_3_-adrenoceptor stimulation.

### Bladder Hypertrophy

Based on pre-specified study protocol, bladder hypertrophy assessed as bladder weight was the primary outcome parameter of our study, with bladder/body weight as a secondary, exploratory parameter. Given that the extent of end-of-study glucose elevation differed markedly between the two batches, we decided that pooling at the group level did not make sense. We observed only a small numerical increase in bladder weight in batch 2 (about 10%; not reaching statistical significance), suggesting that mild hyperglycemia does not cause major bladder hypertrophy. A somewhat greater numerical increase in bladder weight was observed in batch 1 (about 35%), but the null hypothesis was not rejected in this batch with a smaller sample size either, making this an inconclusive data set for analysis of bladder weight. This trend prompted us to perform a *post hoc* secondary analysis in which we compared blood glucose and bladder weight based on individual rats. This yielded a correlation of moderate strength (r^2^ = 0.2013), suggesting that variability of glucose levels between animals could mathematically account for about 20% of the variability in bladder weight. Although bladder hypertrophy is a highly consistent feature of T1DM models (Arioglu Inan et al., 2018), the presence and the reported extent of bladder hypertrophy may vary widely between T2DM models (Ellenbroek et al., 2018). Interestingly, a comparison of blood glucose and bladder weight at the group level across all reported T1DM and T2DM models yielded a correlation coefficient (r^2^ = 0.1725) similar to that we observed within our study at the individual animal level. Although it would be premature to decide the relative role of diabetes-induced polyuria in bladder enlargement, these correlation analyses support a partial but not an overwhelming role of polyuria. This idea is further supported by our finding that treatment of dapagliflozin in the absence of hyperglycemia was associated with increased urine output and concomitantly an increase in bladder weight by about 15% (statistically significant in a *post hoc* analysis).

### Bladder Contraction and Relaxation

Surprisingly, little is known on contractile responses of the diabetic bladder in vitro, particularly in T2DM and/or upon anti-diabetic treatment (Ellenbroek et al., 2018). Therefore, we have used several indicators of contractile and relaxant responses. Contractile peak and plateau responses to carbachol and KCl, regardless whether normalized for strip length or weight, were comparable among groups. Such comparable responses were found in both batches of our study, i.e., most likely true, irrespective of extent of glucose elevation. Accordingly, treatment with dapagliflozin caused no major change in either controls or HFD/STZ animals. In contrast, maximum carbachol responses normalized for KCl-induced contractions were increased in another T2DM model, in 12- and 70-week-old Goto-Kakizaki rats (Saito et al., 2008); however, these data are difficult to interpret as the authors did not report the KCl response. Also, using Goto-Kakizaki rats, others found KCl responses to be similar to those in euglycemic rats, whereas contractions in response to carbachol were similar in both strains at 10 weeks of age and reduced in diabetic animals at 46 weeks, regardless whether normalized for KCl responses (Aizawa et al., 2013). In another T2DM model, Zucker diabetic fatty rats, contractile responses to KCl, carbachol, and bethanechol were largely unchanged (Kendig et al., 2016). In the T1DM model of STZ-injected rats, contractile responses to carbachol were increased, and this was at least partly mimicked in sucrose-fed rats to control for the increased diuresis (Latifpour et al., 1991). Other investigators confirmed increased responses to a muscarinic agonist in one (Longhurst et al., 1991) but not in a subsequent study (Longhurst et al., 2004). When responses were increased, this was normalized by treatment with insulin (Longhurst et al., 1991). An increased density of muscarinic receptors was shown as a possible underlying mechanism of increased responses, but this was only partly normalized upon treatment with insulin (Fukumoto et al., 1994). In STZ-injected mice, increased contractile response to carbachol and to extracellular field stimulation were observed, but this difference was not detected in toll-like receptor 4 knock-out mice (Szasz et al., 2016). Thus, increased, unchanged, and decreased contractile responses have been reported across multiple animal models of diabetes, but it remains unclear whether such differences reflect those in model, species, or age or simply reflect limited robustness of findings.

Even fewer data are available on relaxant responses of the diabetic bladder in vitro, particularly in T2DM and/or upon anti-diabetic treatment (Ellenbroek et al., 2018). Relaxation responses to isoprenaline, the β_2_-adrenoceptor agonist fenoterol, the β_3_-adrenoceptor agonist CL 316,243, and the direct adenylyl cyclase stimulator forskolin were comparable across all four treatment groups of both batches of the present study. Responses in dapagliflozin-treated animals were similar to those in the control and HFD/STZ rats. In STZ-injected rats, relaxation responses to isoprenaline were increased, and this was at least partly mimicked by sucrose feeding (Latifpour et al., 1991). This was accompanied by an increased density of β-adrenergic binding sites in diabetic but not sucrose-fed rats. Another study in the same model did not confirm changes of relaxation induced by isoprenaline or noradrenaline (Longhurst et al., 2004). As with contractile responses, it appears premature to decide whether in vitro relaxation responses are altered in diabetes; if so, it remains unclear whether this may be specific for a given diabetes model.

### Conclusions

In conclusion, mild hyperglycemia was not associated with enlargement or dysfunction of the urinary bladder in our rat model. Pilot data based on limited sample size and statistical power under conditions closer to T2DM also did not detect major hypertrophy or dysfunction. Although the previous literature (as reviewed in [Arioglu Inan et al., 2018; Ellenbroek et al., 2018]) showed various degrees of bladder hypertrophy and dysfunction in animals models of T2DM, our study is the first on mild hyperglycemia. Correlations between glucose level (a proxy for polyuria once the renal glucose threshold is exceeded) and bladder weight at the group level (Arioglu Inan et al., 2018; Ellenbroek et al., 2018) and at the individual animal level in this study as well as the effect of dapagliflozin on diuresis and bladder weight support the view that polyuria may contribute to development of bladder hypertrophy in diabetes. However, the effect of T2DM on bladder weight is highly heterogeneous across models (Ellenbroek et al., 2018), making it difficult to determine whether findings from a single study, such as ours, are representative for T2DM in general or, at least partly, reflect specific aspects of a model. Our study appears to be the first report on effects of an oral anti-diabetic medication in clinical use related to urinary bladder hypertrophy and function. Although this was not informative in the present study in the absence of bladder hypertrophy and dysfunction, it will be important in those models of T2DM exhibiting alterations of bladder structure and function.

## Data Availability

The raw data supporting the conclusions of this manuscript will be made available by the authors, without undue reservations, to any qualified researcher.

## Ethics Statement

Our study has an ethical approval provided by the local animal welfare committee of Ankara University (2015-4-82, 2017-9-84, 2017-23-188).

## Author Contributions

MM and EA-I designed the study. ZEY, BRE, IK, and AEM performed the experiments. ZEY, BRE, and MM made statistical analysis. MM and EA-I wrote the manuscript. ZEY, MM, and EA-I revised the final draft of the manuscript.

## Funding

This work was funded in part by grants from Ankara University (BAP-16L0237006), the Scientific and Technological Research Council of Turkey (TUBITAK SBAG-115S564), and Deutsche Forschungsgemeinschaft (Mi 294/8-1). ZEY has been supported in part by grants from TUBITAK (TUBİTAK-2211/A) and the Erasmus program of the European Union.

## Conflict of Interest Statement

MM is a consultant and shareholder of Velicept Therapeutics and a consultant of Astellas, companies developing and/or marketing β_3_-adrenoceptor agonists for the treatment of overactive bladder.

The remaining authors declare that the research was conducted in the absence of any commercial or financial relationships that could be construed as a potential conflict of interest.
