# Supplementary material for: Urinary Bladder Weight and Function in a Rat Model of Mild Hyperglycemia and Its Treatment With Dapagliflozin
Source: Front Pharmacol. 2019 Aug 16;10:911. doi: 10.3389/fphar.2019.00911 (PMC6706456; doi:10.3389/fphar.2019.00911)

Online supplement to

Urinary bladder weight and function in a rat model of mild hyperglycemia and its treatment with dapagliflozin

Zeynep Elif Yesilyurt, Betül Rabia Erdogan, Irem Karaomerlioglu, Ayhanim Elif Müderrisoglu, Martin Christian Michel, Ebru Arioglu-Inan

Supplemental Table 1: Physiological parameters of animals of batch 1 (pilot study) at study end. Data are means ± SD of 6 rats. Bladder weight did not differ significantly between diabetic and control animals (null hypothesis not rejected).

	Control	Dapagliflozin	Diabetic	Treated diabetic
Blood glucose, mg/dl	112±6	106±10	401±95	174±18
Body weight, g	487 ± 35	459 ± 10	395 ± 33	393 ± 39
Bladder weight, mg	152± 14	180 ± 24	207 ± 84	193 ± 33
Bladder/body weight, g/100 g	0.31 ± 0.03	0.39 ± 0.05	0.53 ± 0.25	0.50 ± 0.09

Supplemental Table 2: Contractile responses of bladder strips of animals of batch 2. Data are means ± SD of 10 rats, except for diabetic group (n = 9) with 2-4 strips being measured per rat.

	Control	Dapagliflozin	Diabetic	Treated diabetic
KCl peak, mN/mg	280±99	276±56	393±210	299±56
Carbachol peak pEC_{50}	6.00±0.20	6.04±0.24	6.20±0.47	5.99±0.32
Carbachol peak E_{max}, mN/mg	491±105	568±117	726±398	620±108
Carbachol plateau, mN/mg	148±42	159±33	218±128	157±42

Supplemental Table 3: Contractile responses of bladder strips of animals of batch 1 (pilot study). Data are means ± SD of 6 rats with 2-4 strips being measured per rat.

	Control	Dapagliflozin	Diabetic	Treated diabetic
KCl peak, mN/mm	267±82	299±92	276±93	291±75
KCl peak, mN/mg	237±68	238±48	219±54	253±72
Carbachol peak pEC_{50}	6.00±0.17	6.01±0.11	6.01±0.17	6.11±0.22
Carbachol peak E_{max}, mN/mm	521±122	586±119	565±162	506±74
Carbachol peak E_{max}, mN/mg	466±80	478±120	444±64	456±103
Carbachol plateau, mN/mm	177±57	177±27	176±46	167±31
Carbachol plateau, mN/mg	156±36	151±30	148±48	146±25

Supplemental Table 4: Relaxant responses of bladder strips in batch 1 (pilot study). Data are means ± SD of 6 rats with 1 strip being measured per rat for the β-adrenoceptor agonists and the mean of 1-3 strips for forskolin. Maximum relaxation responses (E_{max}) and those to 10 µM forskolin are expressed as % of tone, i.e. force prior to addition of first agonist concentration ("carbachol plateau", see Table 2 in main manuscript)

	Control	Dapagliflozin	Diabetic	Treated diabetic
Isoprenaline				
pEC_{50}	6.9±0.2	7.0±0.2	6.9±0.2	6.9±0.4
E_{max}	52±21	51±11	58±4	54±8
Fenoterol				
pEC_{50}	5.7±1.1	5.4±0.2	5.4±0.8	5.6±0.8
E_{max}	50±27	45±17	54±13	49±7
30 µM	54±24	48±16	59±6	55±4
CL 316,243				
pEC_{50}	7.4±0.2	7.5±0.1	6.8±1.2	7.5±0.4
E_{max}	74±10	75±7	70±16	70±8
Forskolin				
10 µM	-29±28	-34±12	-17±12	-23±6

Supplemental Figure 1: Chemical structure of dapagliflozin.

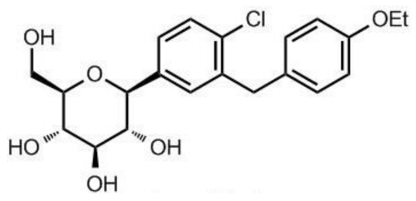

Supplemental Figure 2: Time course of blood glucose and body weight of control rats (black symbol), control rats treated with dapagliflozin (blue symbols), HFD + low-dose STZ-treated rats red symbols) and HFD + low-dose STZ + dapagliflozin-treated rats (green symbol). Each data point represents mean ± SD of 10 rats (9 for HFD + low-dose STZ group). As start of treatment with dapagliflozin differed slightly between animals, data are shown relative to the individual start of treatment (week 18-19).

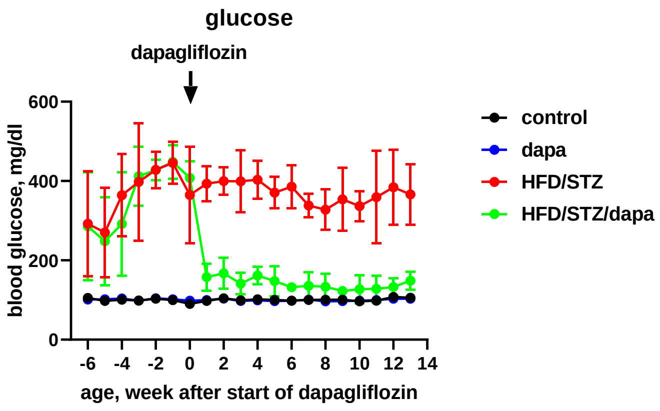

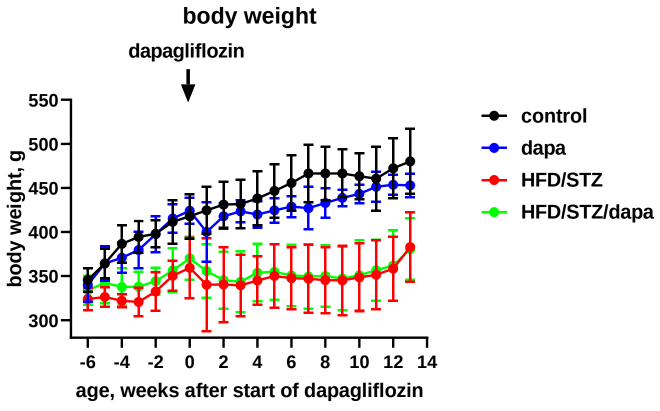

Supplement: Supplementary file 1 [file DataSheet_1.pdf]
